# Identification of targets for rational pharmacological therapy in childhood craniopharyngioma

**DOI:** 10.1186/s40478-015-0211-5

**Published:** 2015-05-21

**Authors:** Jacob M. Gump, Andrew M. Donson, Diane K. Birks, Vladimir M. Amani, Karun K. Rao, Andrea M. Griesinger, B. K. Kleinschmidt-DeMasters, James M. Johnston, Richard C. E. Anderson, Amy Rosenfeld, Michael Handler, Lia Gore, Nicholas Foreman, Todd C. Hankinson

**Affiliations:** Department of Pharmacology, University of Colorado Anschutz Medical Campus, Aurora, CO 80045 USA; Departments of Pediatrics, University of Colorado Anschutz Medical Campus, Aurora, CO 80045 USA; Department of Neurosurgery, University of Colorado Anschutz Medical Campus, Aurora, CO 80045 USA; Departments of Pathology and Neurology, University of Colorado Anschutz Medical Campus, Aurora, CO 80045 USA; Department of Neurosurgery, Children’s Hospital Alabama, Birmingham, AL 35233 USA; Department of Neurological Surgery, Columbia University Medical Center, New York, NY 10032 USA; Center for Cancer and Blood Disorders, Phoenix Children’s Hospital, Phoenix, AZ 85016 USA; Department of Neurosurgery, Children’s Hospital Colorado, Aurora, CO 80045 USA; Center for Cancer and Blood Disorders, Children’s Hospital Colorado, Aurora, CO 80045 USA; Adult and Child Center for Health Outcomes Research, University of Colorado Anschutz Medical Campus, Aurora, CO 80045 USA

## Abstract

**Introduction:**

Pediatric adamantinomatous craniopharyngioma (ACP) is a histologically benign but clinically aggressive brain tumor that arises from the sellar/suprasellar region. Despite a high survival rate with current surgical and radiation therapy (75–95 % at 10 years), ACP is associated with debilitating visual, endocrine, neurocognitive and psychological morbidity, resulting in excheptionally poor quality of life for survivors. Identification of an effective pharmacological therapy could drastically decrease morbidity and improve long term outcomes for children with ACP.

**Results:**

Using mRNA microarray gene expression analysis of 15 ACP patient samples, we have found several pharmaceutical targets that are significantly and consistently overexpressed in our panel of ACP relative to other pediatric brain tumors, pituitary tumors, normal pituitary and normal brain tissue. Among the most highly expressed are several targets of the kinase inhibitor dasatinib – *LCK, EPHA2* and *SRC*; *EGFR* pathway targets – *AREG*, *EGFR* and *ERBB3*; and other potentially actionable cancer targets – *SHH*, *MMP9* and *MMP12*. We confirm by western blot that a subset of these targets is highly expressed in ACP primary tumor samples.

**Conclusions:**

We report here the first published transcriptome for ACP and the identification of targets for rational therapy. Experimental drugs targeting each of these gene products are currently being tested clinically and pre-clinically for the treatment of other tumor types. This study provides a rationale for further pre-clinical and clinical studies of novel pharmacological treatments for ACP. Development of mouse and cell culture models for ACP will further enable the translation of these targets from the lab to the clinic, potentially ushering in a new era in the treatment of ACP.

## Introduction

Adamantinomatous craniopharyngioma (ACP) is the most common non-neural brain tumor with an incidence of approximately 1.9 cases/million patient-years in children [1–3]. Due to its sensitive sellar/suprasellar location and propensity to form large cysts, ACP often compresses and damages vital structures of the pituitary, hypothalamus and visual apparatus. Although seemingly well-demarcated on neuroimaging studies, histology reveals finger-like protrusions extending into neighboring visual and hypothalamic structures, eliciting tissue damage and gliosis [4]. This propensity to invade adjacent structures, in addition to the difficult surgical location, often precludes total resection in order to avoid the significantly increased risk of visual and hypothalamic damage associated with attempts to completely remove the tumor [5–8]. The current standard of subtotal resection followed by radiation reduces some of the morbidity, however, it makes recurrence relatively common, even after apparently successful primary therapy. Outcomes after recurrence are poorer, with significantly higher mortality and morbidity than after primary treatment [9–11].

While conservative surgery and radiation confer low mortality, the morbidity for survivors is still unacceptably high. Variable morbidities are associated with ACP but include endocrine, neurological, vascular, psychological and visual deficits [12]. As a result, ACP has been associated with the lowest quality of life (QoL) scores of any pediatric brain tumor [13]. Lifelong care is necessary for most childhood craniopharyngioma patients and ACP and is considered by many to be a chronic disease [14]. The introduction of rational therapy to treat craniopharyngioma could drastically reduce the morbidity associated with both the primary disease and current treatments by reducing the extent of resection and/or reducing or eliminating the need for subsequent radiation. Such a paradigm change in ACP treatment is critical to improving long term QoL for patients with this debilitating disease.

Progress regarding our understanding of the biological drivers of ACP growth has been slowed by the relative rarity of the tumor and the recalcitrance of ACP cells to laboratory growth. Lack of knowledge of the underlying biology, combined with the clinical complexity of ACP have led to an absence of standard systemic antitumor therapies. Few attempts to remedy this deficit have been made, in part because current therapy has acceptable survival outcomes. Nevertheless, substantial progress has been made recently through tissue banking collaborations and “omics” approaches.

Virtually all craniopharyngiomas in childhood are of the adamantinomatous type (ACP), contrasting with adults in whom up to 10 % of craniopharyngiomas are papillary, and are now known to be driven by BRAF V600E mutations [15]. The only known genetic alterations in adamatinomatous craniopharyngioma (ACP) are point mutations in exon 3 of *CTNNB1* that lead to β-catenin accumulation and upregulation of downstream target gene expression. While the reported frequency of *CTNNB1* sequence alterations ranges from 16–100 % [16–19], Brastianos and colleagues [15] recently used whole exome sequencing and mass spectrometric genotyping to identify *CTNNB1* mutations in 92–96 % of ACP. It is likely, however, that genetic, epigenetic or other biological factors in addition to *CTNNB1* mutation contribute to the pathogenesis of ACP. For instance, Larkin and colleagues [20] described 2 tumors that harbored alterations in both *CTNNB1* and *BRAF.* Furthermore, ACP tumors with *CTNNB1* mutation contain cells that do not demonstrate intranuclear β-catenin accumulation [21] and it has been suggested that some of the cells that comprise the tumor may not actually be *CTNNB1* mutant “tumor” cells at all [22]. EGFR pathway activation has also recently been identified as a driver of migration and growth using *in-vitro* and xenotransplant models of ACP, supporting the testing of EGFR targeted therapies [23, 24]. In addition, through an embryonic mouse model of human ACP, the role of pituitary stem cells in ACP tumorigenesis is being explored [22, 25, 26].

The recent identification of *BRAF* mutations in papillary craniopharyngioma changes the paradigm in treating this (primarily adult) tumor because of the availability of BRAF V600E-specific inhibitors. By contrast, the identification of β-catenin/Wnt signaling as a driver of adamantinomatous craniopharyngioma (ACP) is of little use in guiding therapy because inhibitors of Wnt signaling downstream of β-catenin/TCF/LEF are not yet clinically viable [27]. Global gene expression analysis is therefore critical for determining the epigenetic effect of aberrant β-catenin driven transcription in ACP in order to find targets for rational therapy [22, 28].

## Materials and methods

### Tumor samples

A total of 15 ACP tumor samples were included in this study. Eleven specimens were from patients who underwent surgical procedures at Children’s Hospital Colorado, from 1995 through 2014. Tumor samples were collected at the time of surgery and snap frozen in liquid nitrogen or fixed in formalin and paraffin embedded. Additional specimens were contributed by the University of Alabama, Columbia University and Phoenix Children’s Hospital. The median age of this cohort was 7 years (range 0 to 18 years) (Table 1). Purity of ACP tumor samples was determined by histological analysis using hematoxylin and eosin staining in addition to immunostaining for β-catenin. A further 176 samples of other primary tumors and a variety of normal cerebral tissues were used for comparative purposes. This cohort included samples from the spectrum of pediatric and adult brain tumor types (20 atypical teratoid/rhabdoid tumor (AT/RT), 5 choroid plexus papilloma (CPP), 46 ependymoma (EPN), 12 glioblastoma (GBM), 22 medulloblastoma (MED), 9 meningioma (MEN), 15 pilocytic astrocytoma (PA), 13 primitive neuroepithelial tumor (PNET)) and other peripheral pediatric solid tumors (6 malignant peripheral nerve sheath tumors (MPNST), 8 rhabdomyosarcoma (RMS)). Specimens were classified according to WHO international histological tumor classification. Normal pediatric brain samples from a variety of anatomic sites were obtained during routine epilepsy surgery or autopsy at Children’s Hospital Colorado. All samples were obtained in compliance with internal review board regulations (COMIRB #95-500 and #09-0906).

**Table 1:** β-catenin and BRAF mutational status of tumor and age of 15 ACP patient cohort used in transcriptome study

UPN	β-Catenin	BRAF	Age at Dx
411	S33F	WT	2
463	S37F	WT	4
598	S37C	WT	18
646	D32N	WT	7
673	S37F	WT	2
740	S33F	WT	12
802	WT	WT	7
883	WT	WT	6
956	S37C	WT	9
980	D32N	WT	13
1000	S45F	WT	0
9109	WT	WT	9
9201	T41I	WT	-
9202	T41I	WT	-
9302	WT	WT	-

### Nucleic acid extraction, amplification and microarray preparation

RNA from all surgical specimens was extracted, amplified, labeled and hybridized to Affymetrix HG-U113 plus 2 microarray chips (Affymetrix, Santa Clara, CA, USA) according to manufacturer’s instructions and as described previously [29]. SNaPshot analysis for *CTNNB1* and *BRAF* mutations was performed at the University of Colorado Pathology Core per manufacturer’s instructions and as described previously [22, 30]. Patient characteristics, including presence or absence of *CTNNB1* mutations by SNaPshot analysis, are shown in Table 1. SNaPshot analysis was also used to examine *BRAF* mutational status (Table 1), specifically *BRAF* V600E, which was recently identified in papillary craniopharyngioma [20]. This mutation was not found in any ACP sample tested.

### Microarray data analysis

Data analysis was performed in R (http://www.r-project.org), using packages publicly available through Bioconductor (http://www.bioconductor.org). As a first step, the scanned microarray data were background corrected and normalized using the gcRMA algorithm [31] resulting in log 2 gene expression values. Publically available microarray CEL file data were obtained for 9 normal pituitary and 14 pituitary adenoma samples from GEO (GSE26966); these samples were chosen because they were processed in the same manner as ours and through the same microarray core lab [32]. These were combined with the ACP, other tumor, and normal brain cohort as detailed above. Multiple probesets for a gene were then collapsed to 1 entry per gene, based on the mean best-expressed probeset for that gene. Hierarchical clustering was performed using the normalized gene expression data. Distances based on Spearman correlations were calculated for input to an agglomerative algorithm with use of complete linkage, as implemented in the Bioconductor hclust function. These microarray data have been deposited in the National Center for Biotechnology Information Gene Expression Omnibus (GEO) database [33] and are publicly accessible through GEO Series accession number GSE68015 (http://www.ncbi.nlm.nih.gov/geo/query/acc.cgi?acc=GSE68015).

Differential gene expression between ACP and multiple other tumor and normal tissue types was calculated using the Bioconductor limma function [34]. Data were filtered before input to eliminate genes not expressed in any samples or that showed only limited variance across samples. The limma function performs pair-wise comparisons between a target group and each of the other user-defined groups in the dataset. It uses an Empirical Bayes approach to calculate a moderated t-statistic and calculates a false discovery rate (FDR) that accounts for multiple testing both within and across groups. The unique molecular signature of ACP was defined as genes that showed significant differential expression in all of the pair-wise comparisons (FDR ≤0.1 and mean fold difference ≥1.5) and for which the mean difference was in the same direction when compared with all of the other clusters (i.e., either all upregulated or all downregulated in all of the comparisons with respect to ACP).

In further analyses, individual tumor gene expression of select genes involved in putative ACP development/biology were extracted from the normalized dataset. For these comparisons, normalized hybridization intensity values for a selected gene for individual ACP samples were presented as fold-difference relative to the average of all other tumor and normal samples.

Data analysis using hierarchical clustering, NIH Database for Annotation, Visualization, and Integrated Discovery (DAVID) bioinformatics tools has been previously described [29]. Functional analysis of genes was performed with DAVID using the Gene Ontology, Protein Analysis Through Evolutionary Relationships (PANTHER) Biological Process and KEGG databases [35–37]. ACP upregulated genes were compared to known targets of FDA approved and other oncology drugs that had reached clinical trial stage, as published in the literature and compiled in Ingenuity’s KnowledgeBase (Ingenuity Systems, www.ingenuity.com). Oncology drug target gene expression in individual ACP samples (n = 15) was compared to all other tumor and normal tissue combined and assigned percentile scores. Drug target genes that were consistently highly expressed (>66^th^ percentile) in all ACP samples were identified, and then ranked according to the highest average percentile expression.

### Western blot validation of functional isoforms of putative drug targets

Protein levels of a selection of putative drug target genes in ACP were examined by Western blot analysis to validate the results of microarray analysis. Snap frozen tumor samples were homogenized in RIPA buffer (Sigma) supplemented with protease and phosphatase inhibitors (Roche). This study utilized 6 ACP samples and 3 each of AT/RT, EPN, GBM, MED, PA and normal brain (obtained from autopsy material). Proteins samples (30 μg) were resolved on a 26 well Criterion Gel (BioRad) and transferred to Immobilon PVDF membrane (Millipore). Membranes were probed with antibodies to SHH (Millipore #06-1106; 1:1000), MMP9 (Cell Signaling #3852; 1:1000), MMP12 (R&D Systems #AF917; 1:1000) and Actin (Cell Signaling #12262; 1:10,000).

## Results

### Identification of potential drug targets and ACP signature genes

**Fig. 1:**
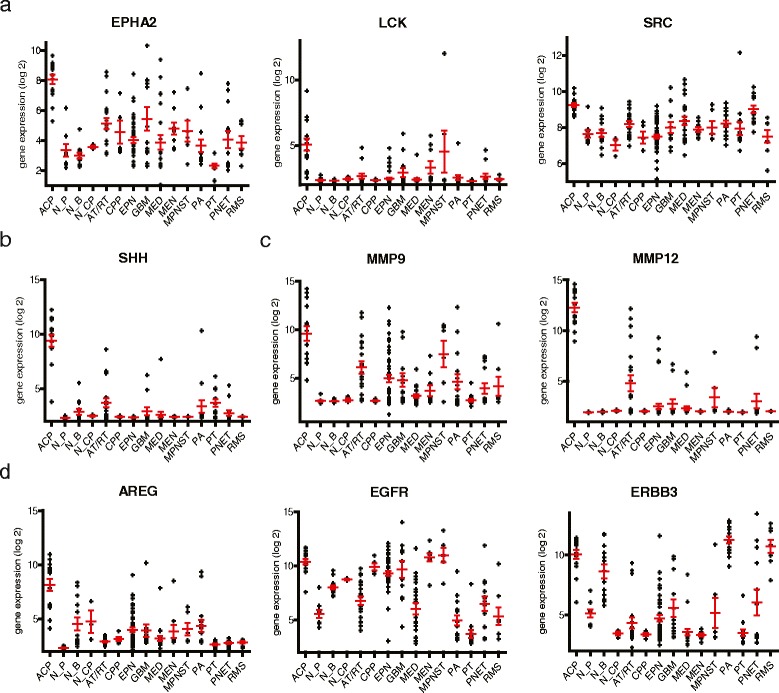
Gene expression of potential targets for therapeutic intervention in ACP. Expression of the indicated genes in ACP relative to a broad range of pediatric and adult brain tumor types: atypical teratoid/rhabdoid tumor (AT/RT), choroid plexus papilloma (CPP), ependymoma (EPN), glioblastoma multiforme (GBM), medulloblastoma (MED), meningioma (MEN), pilocytic astrocytoma (PA), primitive neuroectodermal tumor (PNET); peripheral pediatric solid tumors: malignant peripheral nerve sheath tumors (MPNST), rhabdomyosarcoma (RMS); in addition to malignant (pituitary adenoma) and normal pituitary (PT and N_P respectively); and normal brain and choroid plexus (N_B and N_CP respectively). Dasatinib targets, *LCK* (lymphocyte-specific protein tyrosine kinase), *EPHA2* (ephrin type-A receptor 2) and *SRC* (SRC proto-oncogene, non-receptor tyrosine kinase) **(a)**. Sonic hedgehog homolog **(b)**. Matrix metalloproteases 9 & 12 **(c)**. EGF pathway genes **(d)**. Values are expressed as log2 gene expression. Horizontal red bars represent the mean, and error bars represent standard error of the mean (SEM)

**Fig. 2:**
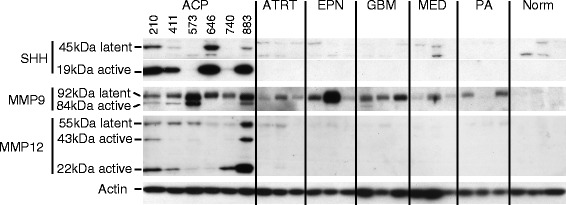
Overexpression of active protein isoforms for SHH, MMP9 and MMP12 in ACP relative to other common pediatric brain tumors and normal brain. Western blot analysis was used to determine latent preforms and cleaved active isoforms of the indicated proteins. (Abbr: AT/RT, atypical teratoid/rhabdoid tumor; EPN, ependymoma; GBM, glioblastoma; MED, medulloblastoma; PA, pilocytic astrocytoma; Norm, normal brain)

The β-catenin and BRAF mutational status of each tumor sample is presented in Table 1. To explore the clinical relevance of our human pediatric ACP transcriptomic data, we screened the ACP signature for upregulation of genes associated with potential oncological drug targets for the treatment of these tumors. Genes with high expression in ACP samples (n = 15) versus all other normal and neoplastic tissue samples (n = 195) were compared to known targets of oncology drugs as published in the literature and compiled in IPA KnowledgeBase. This approach identified 13 drug target transcripts that were consistently overexpressed greater than the 66^th^ percentile in ACP (Table 2). Three of the 13 consistently elevated drug target genes, *LCK* (lymphocyte-specific protein tyrosine kinase) (5.5 fold change (FC) in ACP versus all other samples combined, p = 8.2 × 10^−16^), *EPHA2* (ephrin type-A receptor 2) (FC = 16.6, p = 6.8 × 10^−16^) and *SRC* (SRC proto-oncogene, non-receptor tyrosine kinase) (FC = 2.5, p = 3.7 × 10^−7^), are targets of a single FDA-approved drug, dasatinib (Fig. 1a). Other combinations of genes overexpressed by ACP that could be targeted by a single drug were identified. *SHH* (sonic hedgehog homolog) was shown to be highly expressed (FC = 96.9, p = 1.5 × 10^−48^) by ACP in this study (Fig. 1b), which is consistent with a recently developed mouse model of ACP [25] and is the focus of oncology drug development for a number of tumor types. SHH is translated as an inactive precursor 45 kDa protein that is cleaved to generate an active 19 kDa isoform. As a putative drug target in ACP, we measured the levels of precursor and active isoforms for SHH by Western blot analysis. Isoform levels were compared to a panel of other pediatric brain tumor types and normal brain. This revealed that although the inactive precursor is expressed broadly across all tumor types, the active isoform is present at high levels only in ACP (Fig. 2). ACP was also found to overexpress *MMP9* (FC = 41.0, p = 9.8 × 10^−14^) and, more strikingly, *MMP12* (FC = 819.7, p = 3.0 × 10^−49^) (Fig. 1c), which are both inhibited by AZD1236, a drug originally tested as a treatment for chronic obstructive pulmonary disease, but more recently investigated as an antitumor agent. Like SHH, MMP9 and 12 are translated as inactive precursor proteins (92 and 55 kDa respectively) that are cleaved to generate active isoforms (84 and 43/22 kDa, respectively). MMP isoform levels were compared to a panel of other pediatric brain tumor types and normal brain. The latent precursor of MMP9 was identified in all tumor types but not normal brain, whereas the active isoform was only observed in ACP (Fig. 2). Both the active and inactive isoforms of MMP12 were restricted to ACP (Fig. 2). ACP additionally overexpressed *AREG* (FC = 20.9, p = 4.8 × 10^−17^), *EGFR* (FC = 7.6, p = 6.0 × 10^−5^) and *ERBB3* (FC = 22.8, p = 4.2 × 10^−8^) (Fig. 1d). Each of these is a target of the numerous EGFR/ERBB pathway inhibiting drugs (cetuximab, erlotinib, lapatinib).

**Table 2:** Top 20 therapeutic target genes overexpressed in ACP compared to normal brain tissues and a variety of CNS and peripheral malignancies

Symbol	Gene name	Average percentile	# of ACP >66th %-ile	Therapeutic agents	Fold	p-Value
MMP12	matrix metallopeptidase 12	99.4	15	AZD1236	820	3.03E-49
SHH	sonic hedgehog homolog	98.9	15	Erismodegib, Vismodegib	96.8	1.48E-48
IL2RB	interleukin 2 receptor, beta	96.7	15	Denileukin diftitox	12.4	4.87E-23
LCK	lymphocyte-specific protein tyrosine kinase	95.2	15	Dasatinib, Pazopanib	5.50	8.24E-16
EPHA2	EPH receptor A2	95.1	15	Dasatinib, Regorafenib	16.6	6.8E-16
AREG	Amphiregulin	93.9	15	Cetuximab	20.8	4.82E-17
PIK3CD	phosphoinositide-3-kinase, catalytic, delta	93.8	15	Idelalisib	6.79	3.55E-10
IL6R	interleukin 6 receptor	91.9	15	Siltuximab	6.13	7.06E-8
MMP9	matrix metallopeptidase 9	91.6	15	AZD1236	41.0	9.79E-14
EPCAM	epithelial cell adhesion molecule	90.7	15	Tucotuzumab celmoleukin	87.4	2.01E-11
SRC	v-src sarcoma (Schmidt-Ruppin A-2) viral oncogene homolog	89.9	15	Bosutinib, Dasatinib	2.47	3.71e-7
MUC1	mucin 1	89.3	15	HuHMFG1	6.30	1.27E-7
ERBB3	v-erb-b2 erythroblastic leukemia viral oncogene homolog 3	86.7	15	Lapatinib	22.8	4.19E-08
TNFSF11	tumor necrosis factor (ligand) superfamily, member 11	91.7	14	Denosumab	5.17	4.28E-8
MAPK14	mitogen-activated protein kinase 14	91.3	14	Regorafenib	2.65	2.49E-8
PTGS2	prostaglandin-endoperoxide synthase 2	85.6	14	Lenalidomide	10.5	9.23E-7
RRAS	related RAS viral (r-ras) oncogene homolog	85.4	14	Sorafenib	3.61	2.32E-5
PSMB1	proteasome (prosome, macropain) subunit, beta type, 1	83.8	14	Carfilzomib	1.45	5.85E-5
EGFR	epidermal growth factor receptor	83.4	14	Cetuximab, Erlotinib, Gefitinib, Lapatinib, etc.	7.57	5.99E-05
CD52	CD52 molecule	86.3	13	Alemtuzumab	8.85	4.51E-12

### Transcriptome microarray clustering analyses

**Fig. 3:**
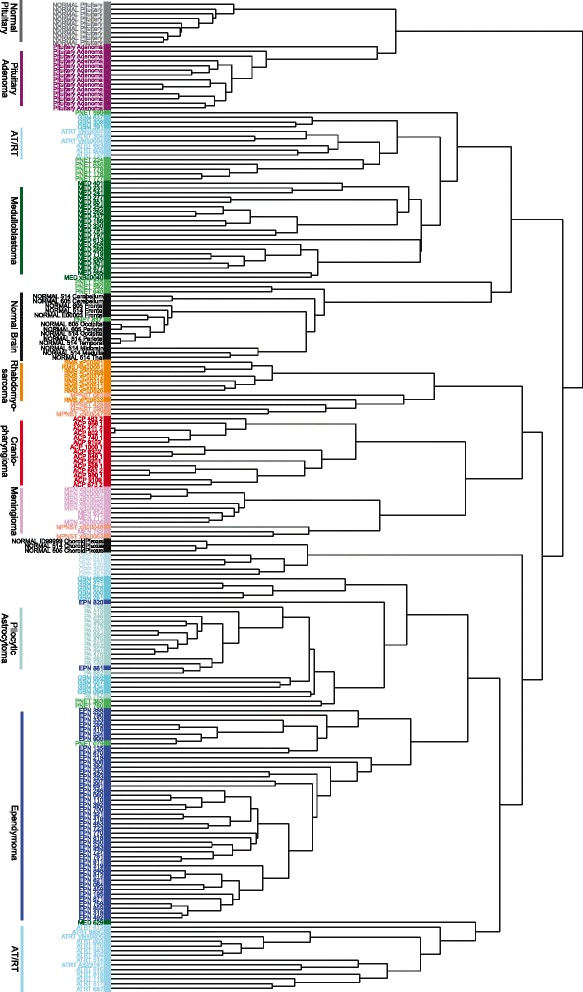
Transcriptome cluster analysis reveals similarities between ACP, meningioma and rhabdomyosarcoma, with no relationship to adult pituitary or pituitary adenoma. Unbiased hierarchical clustering analysis of a panel of craniopharyngioma tumor samples compared to other pediatric tumors, normal brain tissue, pituitary tissue and adult pituitary adenomas. The top 30 % most variant genes, were used to generate the clustering dendrogram above. (AT/RT, atypical teratoid/rhabdoid tumor; CPP, choroid plexus papilloma; MPNST, malignant peripheral nerve sheath tumor; GBM, glioblastoma multiforme)

Unbiased hierarchical clustering of ACP gene expression data with data from the panel of normal and neoplastic CNS samples and some non-CNS pediatric tumor types (as used above) afforded us further insights into the biology of ACP. ACP samples did not group with the cluster containing both normal and neoplastic pituitary (Fig. 3). Surprisingly, ACP formed a distinct cluster within a larger cluster containing MEN, MPNST and RMS, a histologically heterogeneous group of tumors derived from various tissue types. While it is perhaps not unexpected that a non-neural tumor like ACP clusters within this group of non-neural tumors, the fact that this cluster lies within the larger neuro-epithelial tumor and normal brain cluster and not with the pituitary tissues is difficult to interpret. This raises the possibility that ACP may have a completely different origin than has been hypothesized; it is more likely however that it has differentiated in such a way that it shares a convergent expression profile in common with these tumors not due to a common tissue of origin. Further analysis of the gene expression signatures responsible for these groupings as well as comparisons with papillary craniopharyngioma and head and neck cancers may give insight into the nature of these groupings.

### Gene expression signature recapitulates ACP histopathological characteristics

**Fig. 4:**
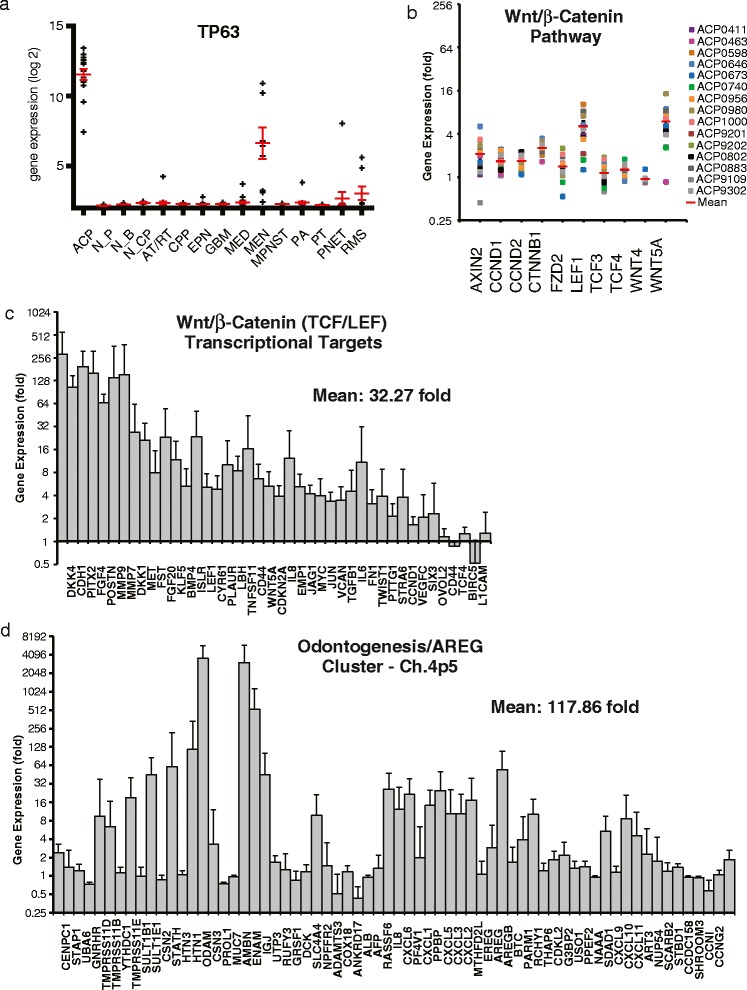
Expression of the indicated genes in pediatric ACP. p63 gene expression in the indicated tumor and normal tissue types **(a)**. Wnt pathway **(b)** and β-catenin (TCF/LEF) target gene expression **(c)**. Genes at chromosome 4p5 locus **(d)**. Values are expressed as log2 **(a)** or as fold-difference of individual ACP samples relative to the average of all other tumor and normal samples **(b-d)**

In a series of pair-wise comparisons (limma) with normal brain from a range of anatomic sites (including pituitary) and other brain (AT/RT, CPP, EPN, GBM, PA, MED, MEN, PNET), pituitary (PT) and peripheral solid tumors of childhood (MPNST, RMS) we identified genes that were overexpressed by ACP in all comparisons (FDR p < 0.05). These 384 ACP signature genes were then examined for enrichment (FDR < 0.005) of gene ontology terms curated by Gene Ontology (biological processes), Panther (biological processes) and KEGG (pathways) databases using DAVID. The majority of ontologic terms were comprised of ectodermal development-related genes (odontogenic, epidermal, epithelial development) (Table 3). Morphologic characteristics of odontogenesis in ACP range from deposits of calcium, which are evident on an x-ray, to development of whole teeth [38, 39]. Specific genes contributing to odontogenesis signature include *DLX2, ODAM, AMBN, AMELX, ENAM, TP63, EDAR, SHH, FGF4*. Epidermal morphology is also a defining histological feature of ACP, which is presumed to develop from nests of epithelium derived from Rathke’s cleft, with further development of non-viable wet keratin “ghost cells” that resemble polyhedral, anucleated corneocytes (final step of keratinocyte differentiation). The ACP genes that were highly expressed within these epidermal ontologies include numerous keratins (*KRT5, KRT13, KRT14, KRT15, KRT16, KRT31, KRT34, KRT85*) and laminins (*LAMA3, LAMC2*). *TP63* expression is also extraordinarily high in ACP (Fig. 4a); as a regulator of odontogenic, epidermal and keratinocyte development, and in regulation of stemness, p63 may play a critical role in ACP development and morphogenesis [40]. We also found that a cluster of odontogenic, cytokine and EGF family proteins at Chromosome 4p5 was highly overexpressed (as much as 4,000 fold) in ACP (Fig. 4d). The genetic relevance of the high levels of expression at this locus is unclear.

**Table 3:** Enriched ontology terms associated with ACP-exclusive genes. Limma identified 384 genes that were exclusively expressed in ACP compared to a normal brain tissues and a variety of CNS and peripheral malignancies. DAVID was used to identify 23 enriched ontologies (FDR < 0.005) that are shown ranked according to fold enrichment. Abbreviation: Ontology ID prefix GO, Gene ontology biological process; BP, panther biological process; HSA, KEGG pathway; FDR, Benjamini false discovery rate adjusted p-value

Ontology term	ID	Fold enrichment	p-Value	FDR
Biomineral formation	GO:31214	13.38	2.53E-07	5.50E-05
Odontogenesis of dentine-containing tooth	GO:42475	11.28	5.50E-06	8.69E-04
Epidermis development	GO:8544	11.06	3.10E-27	2.70E-24
Ectoderm development	GO:7398	10.78	2.62E-28	4.55E-25
Epidermal cell differentiation	GO:9913	9.17	6.27E-08	1.56E-05
Keratinocyte differentiation	GO:30216	9.17	2.74E-07	5.29E-05
Odontogenesis	GO:42476	9.17	5.23E-06	9.09E-04
Epithelial cell differentiation	GO:30855	8.43	5.25E-13	3.04E-10
Skeletal development	BP:201	5.81	7.85E-07	1.96E-05
Epithelium development	GO:60429	5.57	1.46E-10	6.33E-08
Bone development	GO:60348	5.37	1.42E-05	0.00205
Ossification	GO:1503	5.26	4.46E-05	0.00515
Cell structure	BP:286	3.72	2.38E-14	2.98E-12
Cell adhesion-mediated signaling	BP:120	3.66	4.34E-08	1.36E-06
Cytokine-cytokine receptor interaction	HSA:4060	3.38	5.13E-05	0.00557
Skeletal system development	GO:1501	3.28	2.06E-05	0.00274
Cell structure and motility	BP:285	2.85	2.68E-13	1.68E-11
Cell adhesion	GO:7155	2.83	4.39E-08	1.52E-05
Biological adhesion	GO:22610	2.82	4.48E-08	1.30E-05
Cell communication	BP:274	2.46	6.08E-10	2.53E-08
Cell adhesion	BP:124	2.30	1.57E-04	0.00280
Regulation of cell proliferation	GO:42127	2.24	3.69E-05	0.00457
Signal transduction	BP:102	1.43	1.21E-04	0.00251

Wnt pathway genes are expressed homogeneously and not at abnormally high levels in ACP (Fig. 4b), with the exception of the transcription factor (TCF)/lymphoid enhancer-binding factor (LEF) targets *LEF1* and *WNT5A*. However, consistent with the hypothesis that aberrant Wnt signaling (via mutant β-catenin) is responsible for the pathogenesis of ACP, β-catenin/TCF/LEF target genes are overexpressed an average of 32 fold over the other samples (Fig. 4c).

**Fig. 5:**
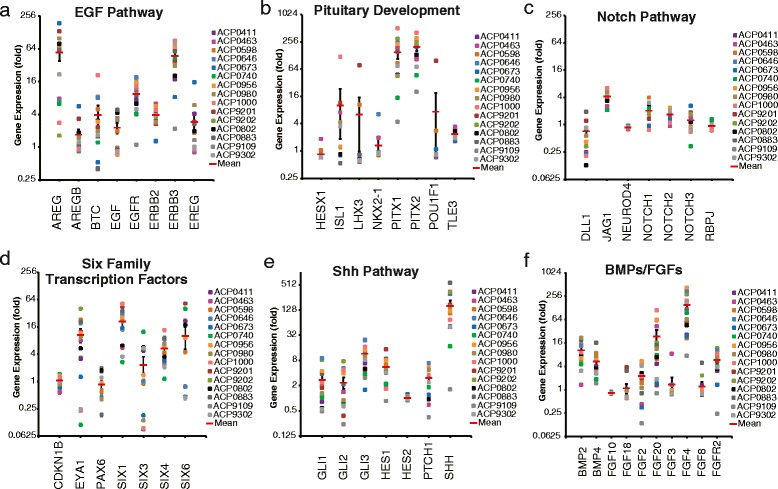
Expression of the indicated developmental and cancer-related genes in individual pediatric ACP samples. Epidermal growth factor (EGF) family genes **(a)**. Genes involved in pituitary development **(b)**. Developmental genes from the Notch **(c)**, Six transcription factor **(d)**, Sonic hedgehog (Shh) **(e)**, Bone morphogenetic protein (BMP) and Fibroblast growth factor (FGF) **(f)** families. Values expressed are fold-difference of individual ACP samples relative to the average of all other tumor and normal samples

The epigenetic profile of ACP may also give some insight into the developmental origins of this disease. Genes involved in pituitary development (Fig. 5a) are not highly expressed with the exception of *PITX1 & 2* which are established TCF/LEF targets. Critical developmental and survival pathway gene expression patterns reveal a potential role for EGFR, Six family transcription factors, Shh and FGFs in ACP pathogenesis (Fig. 5b–f).

## Discussion

Identification of potential therapeutic targets in our transcriptomic analysis confirmed findings of previous studies that had identified SHH and EGFR pathway activity in ACP and provide further evidence that therapies targeting these pathways could be used successfully in treating ACP. Transcriptomic analysis of ACP generated in a mouse model demonstrated high levels of SHH gene expression, suggesting a mitogenic autocrine/paracrine loop [25]. A subsequent study identified upregulation of members of the SHH signaling pathway in human specimens [41]. *In vitro* and xenotransplant model studies have demonstrated that EGFR activation is responsible for driving growth and migration in ACP [23, 24]. The proven clinical utility of EGFR inhibition in the treatment of cancer makes EGFR targeted drugs an attractive approach to ACP treatment. Our identification of high levels of EGFR ligand AREG provide a potential mechanism for EGFR activation in ACP that warrants further exploration. Furthermore, AREG has been implicated as a paracrine/juxtacrine regulator of cell survival in other cancers and epidermal cell types [42]. It has also recently been suggested that ACP may be paracrine in nature (i.e. the *CTNNB1* mutant cells may promote the proliferation of another cell type that actually populates the tumor) [22]. This hypothesis could explain the extensive intratumoral heterogeneity in ACP and perhaps the difficulty we and others have found in obtaining “pure” tumor samples to accurately identify *CTNNB1* mutations [15].

We also identified a number of additional novel potential drug targets. One group, including LCK, EPHA2 and SRC, is targeted by the kinase inhibitor dasatinib. Hundreds of trials are now underway using dasatinib to treat a wide variety of cancers beyond the few for which it currently has FDA approval. A second group of targets we identified are extracellular proteases, of which many were strongly represented in our analysis; two in particular, MMPs 9 and 12, are targeted by the drug AZD1236. MMP12, which we found expressed at very high levels, is a proteolytic factor that may contribute to the significant invasive phenotype that is a hallmark of ACP [43]. Previous studies have attempted to correlate proteinase activity with biological course in ACP [44] including a study that confirmed the presence of MMP9 by immunohistochemistry [45].

The results of this transcriptomic study of human pediatric ACP shed further light on the biology of this tumor, reflect the odontogenic and epithelial characteristics in the pathology of ACP and recapitulate the origins of ACP from oral ectoderm. The origins of ACP have long been the subject of speculation due to the oddity of their morphology and location. Many consider ACP to be a congenital midline developmental malformation, but the (albeit infrequent) occurrence of ACP in late adulthood and variable presentation are difficult to reconcile with this hypothesis. The recognition that β-catenin dysregulation is responsible for ACP have led to two conflicting mouse models for the formation of ACP – in mice an ACP-like tumor can develop from targeted *CTNNB1* mutations in either pituitary oral ectoderm precursors in developing mice embryos or in pituitary stem cells in post-natal mice [46]. The data presented here are consistent with the theory that ACP is a congenital malformation that develops from the improper closure of the craniophyrangeal duct from the oral ectoderm-derived remnants of Rathke’s pouch [46–48] (Table 3). However, these data are not completely inconsistent with the hypothesis that ACP arises from an anterior pituitary stem cell that transdifferentiates toward an oral epithelial phenotype [25, 26]. Further analysis of more ACP samples, including adult ACP, in addition to transcriptomic studies of neuropathologically distinct cell subtypes present within ACP tumors will contribute to our understanding of how these tumors form and perhaps how to better treat them.

## Conclusions

Current standard therapy for ACP is surgery and radiation, both of which lead to high morbidity in this sensitive region of the brain, especially in children, in whom these morbidities become a lifelong and life-altering disease. Intracystic delivery of therapeutic agents (interferon-alpha, bleomycin or Ytrrium^90^) has shown some efficacy in treating ACP [49], but this approach is limited by the requirement of a single cyst in the presenting tumor and requires stereotactic surgery to place a catheter and Ommaya reservoir for delivery. Systemic therapy could more safely and more effectively treat children with ACP. However, progress has been hindered by the absence of *in vitro* or *in vivo* models of this tumor that would enable the unbiased screening of drug libraries. This study forms the basis for further studies with rational therapies for ACP. The recent development of ACP xenotransplants in immune deficient mice [24] will enable pre-clinical testing of these rationally selected targeted therapies, providing further rationale for small studies on efficacy in augmenting surgery and radiation or in treating recurrent ACP. These efforts, combined with further collaborations between centers and consortiums will provide the foundation for a randomized clinical trial using targeted agents to treat ACP in the near future.
